# Pathophysiology and Mechanisms of Saphenous Vein Graft Failure

**DOI:** 10.21470/1678-9741-2022-0133

**Published:** 2022

**Authors:** Gustavo Antonio Guida, Gianni D. Angelini

**Affiliations:** 1 Department of Cardiac Surgery, Bristol Heart Institute, Bristol University, Bristol, United Kingdom

**Keywords:** Coronary Artery Bypass, Coronary Artery Disease, Atherosclerosis, Inhibitors, Hyperplasia, Review.

## Abstract

**Introduction:**

Coronary artery bypass grafting remains one of the best therapies for advanced coronary artery disease. The most used conduit remains the great saphenous vein, which is susceptible to short-term and long-term failure, the result of acute thrombosis, intimal hyperplasia, and late superimposed atheroma. In this review, we present the current findings related to the pathophysiology of vein graft failure.

**Methods:**

A search of three databases - MEDLINE®, Web of Science™, and Cochrane Library - was undertaken for the terms “pathophysiology”, “prevention”, and “treatment” plus the term “vein graft failure”.

**Results:**

The pathophysiology of saphenous graft failure can be classified in three distinct phases - acute thrombosis, intimal hyperplasia, and accelerated atherosclerosis. All these processes start with an underlying histological predisposition of the vein and at the time of harvesting and preparation for grafting. These mechanisms are a result of localized inflammatory and prothrombotic cascades that obey different causes, but ultimately result in the stenosis or occlusion of the vein graft.

**Conclusion:**

The interaction between the different parts of the pathophysiology of vein graft failure is extremely complex and variable. Recent improvements in surgical techniques and secondary pharmaceutical prevention like early aspirin administration and long-term statin treatment have significantly reduced early and late saphenous vein graft failure. However, this continues to be a fascinating area of research with the potential for further improvement for patients and health service provision.

**Table:** 

Abbreviations, Acronyms & Symbols	
CABG	= Coronary artery bypass grafting
EC	= Endothelial cells
ECM	= Extracellular matrix
GSV	= Great saphenous vein
IH	= Intimal hyperplasia
IL	= Interleukin
KLF	= Kruppel-like factor
microRNAs	= Micro ribonucleic acids
NO	= Nitric oxide
PDGF	= Platelet-derived growth factor
SV	= Saphenous vein
VSMC	= Vascular smooth muscle cells

## INTRODUCTION

Coronary artery bypass grafting (CABG) is one of the procedures available to treat coronary artery disease^[^1^]^. One of the most readily available conduits is the great saphenous vein (GSV). However, it suffers from a higher failure rate when compared to arterial grafts^[^2^,^3^]^. This is in part due to the requirement for the vein to adapt to the arterial blood pressure; it has been described that endothelial cells (EC) are sensitive to changes in shear stress, and vascular smooth muscle cells (VSMC) are able to detect changes is pressure^[^4^,^5^]^. The adaptation of the vein to being implanted to the arterial tree is necessary to secure the graft’s longevity, and it does so by developing a degree of wall thickening, also known as arterialization. In some veins, however, the process does not stop, and excessive intimal hyperplasia (IH) will either cause graft failure directly, by obliterating or severely constricting the lumen of the vessel, or indirectly by precipitating accelerated atherosclerosis^[^6^]^.

Research over the last four decades has focused on understanding the pathophysiology of saphenous vein (SV) graft failure to improve treatments capable to increase the longevity of this conduit. The present study presents a current literature review on this topic.

## METHODS

Original research articles and reviews were selected as they related to the pathophysiology, prevention, and treatment of SV graft failure. A literature search was performed using MEDLINE®, Web of Science™, and Cochrane Library. The search was focused on human, translational, *in vitro*, and animal studies.

## RESULTS AND DISCUSSION

### Pathophysiology of SV Graft Failure

This is a complex interaction of several mechanisms and signalling pathways that are activated both locally at the level of the vein’s cells and systemically^[^4^,^7^,^8^]^.

Recent studies have shown that some veins display a degree of IH even before harvesting, while other veins suffer from dilated walls and varicosities, rendering them more susceptible to failure than healthy veins^[^9^]^.

### Acute Thrombosis

The earliest form of SV graft failure is acute thrombosis^[^10^-^12^]^. This phenomenon starts when the vein is harvested, and several factors including ischaemia, mechanical forces, alterations in the pH, and exposure to free oxidizing radicals cause direct damage to both EC and VSMC, sometimes exposing the extracellular matrix (ECM) to the lumen of the vessel^[^6^-^8^,^13^]^. This damaged ECs activate a pro-thrombotic cascade of factors, such as intercellular adhesion molecule 1, vascular cell adhesion protein, selectin, thrombomodulin, and insulin-like growth factor. Simultaneously, there is a reduction of local anti-thrombotic and vasodilatory molecules, like nitric oxide (NO) and prostacyclin^[^6^,^8^,^14^]^. The expression of such factors by the ECs and the exposure of the ECM to the circulation cause activation of platelets and leukocytes^[^8^,^11^,^15^]^. The platelets will mediate thrombus formation by adhering to the ECM or active ECs and generating thrombogenic factors, such as platelet-derived growth factor (PDGF), transforming growth factor β, fibrinogen, fibronectin, and von Willebrand factor^[^7^,^12^,^14^,^16^-^18^]^. Activated platelets also express adhesion molecules, like P selectin and E selectin. Circulating leukocytes, predominantly macrophages, will adhere to the platelets and infiltrate the wall of the vein, secreting pro-inflammatory interleukins both locally and into the circulation^[^8^].^

The abovementioned mechanism results in an imbalance between vasoconstrictors/pro-thrombotic factors and vasodilator/anti-thrombotic factors in favour of the former. This is particularly more prominent where the flow through the vessel is impaired, either because of a technical failure in constructing the anastomosis, or because of a poor run-off in the target coronary^[^12^,^16^]^. In five to 10% of cases, this will generate a local thrombus that will cause early failure of the vein graft^[^12^,^16^]^.

Several studies have identified the use of aspirin as an important therapy to reduce the incidence of early graft failure^[^19^-^21^]^. More recent studies suggest that early and late graft failure rates can be improved by harvesting the vein with minimal manipulation and without distention (preventing mechanical destruction of the endothelium), preserving the pedicle (to reduce the vascular ischemia and increase the availability of NO and prostacyclin), and preserving the vein in a buffered solution before the grafts are constructed (to reduce the damage of the endothelium caused by acidosis)^[^22^-^26^]^.

After this initial phase of endothelial damage and endothelial dysfunction, there is a phase of re-endothelization of the vessel, and *ex-vivo* experimental models proved that new ECs can be seen as early as 36 hours after the vein was harvested, although is likely that *in-vivo* this will take longer^[^6^]^. In some cases, this neoendothelium is dysfunctional and more propense at expressing pro-inflammatory substances^[^18^,^27^]^. The origin of these new ECs is not yet known.

### Intimal Hyperplasia

The next step in the adaptation of the vein to its new environment consists in activation, phenotypic switching, and migration of the VSMC from the medial to the intimal layer of the vein^[^10^,^11^,^13^,^28^]^. This is the process known as IH, and in its physiological state it provides the vein with a more stable intimal layer that will withstand the arterial pressure and shear stress, which is called arterialization of the SV^[^6^]^. This process, however, can instead follow a pathophysiological pathway and being responsible ultimately for the failure of the graft^[^6^]^.

The IH process starts with paracrine interactions between the ECs and the VSMCs; in a healthy state, the interaction between ECs and VSMCs keeps the VSMCs in a quiescent state, via EC-derived homeostatic molecules like NO, which help to regulate the tone of the medial layer and suppress VSMC phenotypic switching to synthetic cells^[^29^-^32^]^. However, similarly as to the mechanism of thrombosis, the dysfunction and destruction of the ECs cause alterations in the interactions between these and the VSMCs. Furthermore, a state of localized inflammation can develop by the creation of positive feedback loops, which induce more inflammation, and, therefore, more endothelial dysfunction^[^4^,^8^,^15^,^33^]^.

In this pro-inflammatory state, ECs activate platelets which secrete cytokines, like interleukin-6 (IL-6), IL-8, thromboxane A2, and tumoral necrosis factor alpha, and growth factors, like PDGF and fibroblast growth factor^[^18^,^33^]^. Activated ECs also produce a particular set of ECM proteins that when detected by the VSMC causes these cells to switch from the quiescent and contractile state to the active and proliferative one^[^29^]^. Another proposed way of communication between ECs and VSMCs is through micro ribonucleic acids (microRNAs), and studies have identified that microRNA-126 resulted in VSMCs increased apoptosis and mitosis^[^30^,^34^,^35^]^.

Internal transcription factors, like Kruppel-like factor 4 (KLF4) and KLF5, and signalling pathways, like p38 mitogen-activated protein kinase (or p38) and nuclear factor kappa-light-chain-enhancer of activated B cells (or NF-κB), within the VSMCs have been identified as responsible for the phenotypic switching of these VSMCs from their quiescent and contractile state to the active and proliferative one^[^4^,^10^,^36^-^41^]^. The up regulation of these transcription factors seems to be linked to reduced expression of VSMCs contractile proteins. These transcription factors and signalling pathways could potentially provide targets for treatment to prevent SV graft failure.

### Accelerated Atherosclerosis

The neointima layer in veins that had been implanted into the arterial circulation is more susceptible to the formation of an atheroma plaque^[^42^]^. This, as with normal atherosclerosis, is a process mediated by local inflammation, infiltration of foam cells, and accelerated lipid uptake within the wall of the vessel^[^6^,^42^]^. In the particular case of the vein, it has been identified that some of the foam cells in the atheroma plaque come from undifferentiated VSMCs rather than macrophages, like the case of arterial atheroma^[^6^]^. The capsule of the atheroma plaque in veins is also softer than that of arteries, therefore, more susceptible to plaque rupture and thrombosis^[^43^]^.

It is thought that like in the case of arterial atherosclerotic risk factor control, lipid lowering agents and anti-platelets agents, such as aspirin, lower the risk of plaque formation and rupture, although further studies looking specifically to veins atherosclerosis are needed to definitively confirm this hypothesis^[^19^-^21^,^44^,^45^]^.

### Prevention of Vein Graft Failure

In order to better understand what we can do to prevent GSV graft failure, we can classify the strategies or interventions in pre-surgery, surgical, and post-surgery.

### Pre-Surgery

Most interventions to prevent GSV graft failure are done during or after surgery, nonetheless, the disease that affects these patients is a form of atherosclerosis and, therefore, the modification of risks factors pre or postoperatively will have an impact on the rate of failure of the grafts^[^9^]^. Furthermore, there is some evidence that poor vein quality correlates with worse long-term outcomes^[^9^,^46^,^47^]^. Some authors even suggest preoperative vein mapping to select the best conduit^[^47^]^. There is clearly a need for larger trials and series investigating the effectiveness of preoperative vein mapping, whilst it is certainly useful for minimising leg wound infections^[^48^]^, it still needs to prove its effectiveness for predicting graft failure.

### Surgical

Several surgical techniques or interventions have been proposed to attempt to reduce GSV graft failure.

One of such interventions that has demonstrated to improve the rates of success has been harvesting the vein with its pedicle and with minimal manipulation^[^24^-^26^,^49^,^50^]^. Proponents of the technique have shown that the perivascular tissue has an important role in interrupting the pathophysiology described above by increasing the local concentration of NO^[^25^]^. This “no-touch” technique also proposes avoiding distention of the vein, thus preventing some degree of endothelial damage, and this was also described separately by previous authors^[^26^,^51^]^.

After the harvesting of the vein and before the vein is implanted, the conduit is often stored in a solution to prevent desiccation, and the solution used varies between surgeons and centres^[^52^]^. Despite the variation, it has been demonstrated that the most important factor is preventing acidosis in the solution by using a buffered solution, either saline or blood^[^52^,^53^]^.

The anastomotic technique and the use of graft quality checks like transit-time flow measurement are useful for preventing the technical failure of the graft, poor anastomotic technique, and poor distal run-off, increasing the turbulence of the flow in the graft, which in turn increases the shear stress on the endothelium, increasing the likelihood of acute thrombosis and, subsequently, graft failure^[^54^-^57^]^.

Another proposed intervention at the time of surgery is to use an external support for the vein graft. The intention is to impose graft symmetry, more laminar flow, and the subsequent reduction of shear stress, and also providing a protective environment for the formation of new adventitia^[^58^]^. Despite some promising initial results, subsequent studies did not manage to replicate them, and further research will be needed if this is to become a regular technique^[^58^-^62^]^.

### Post-Surgery

Apart from modification of atherosclerotic risk factors as mentioned above, the main focus of the post-surgical prevention is the use of pharmacological agents.

For many years, the main drug used to prevent graft failure was aspirin alone^[^20^,^56^]^. However, recently there has been more evidence supporting the use of dual antiplatelets agents and the addition of lipid-lowering drugs, such as statins, for the prevention of GSV graft failure^[^9^,^21^,^45^,^63^]^. Currently, the American Heart Association recommends the use of dual antiplatelets and statins combined in every patient that does not have a contraindication to such treatment^[^44^,^45^]^. The long-term results in population studies of the efficacy of using this medical therapy will be seen in the next five to 10 years, but the initial findings are encouraging.

## CONCLUSION

The pathophysiology of vein graft failure is a complex interaction of several mechanisms, and this manifests in patients who are already suffering from atherosclerosis, making the interaction between these processes even more complex. Nonetheless, continuous research in this area over the last decades has provided us with a more in-depth understanding of the pathophysiology and paved the way to better techniques and treatments that decrease the incidence of SV graft failure.

Despite all we have learned on the process of this condition, much still remains to be learned, and potential therapeutic targets already discovered are still in need of experimental testing. Continuing this line of research may further improve the quality of treatment provided when performing CABG and, therefore, affect morbidity and mortality in the population.

**Table:** 

Authors’ Roles & Responsibilities	
GAG	Substantial contributions to the conception or design of the work; or the acquisition, analysis, or interpretation of data for the work; drafting the work or revising it critically for important intellectual content; final approval of the version to be published
GDA	Substantial contributions to the conception or design of the work; or the acquisition, analysis, or interpretation of data for the work; drafting the work or revising it critically for important intellectual content; final approval of the version to be published
